# Which acute deterioration tools are used in long-term care facilities and how have they been evaluated? A scoping review

**DOI:** 10.1186/s12913-025-12534-x

**Published:** 2025-05-28

**Authors:** Robert O. Barker, Claire H. Eastaugh, Ben Searle, Sheila A. Wallace, Dawn Craig, Barbara Hanratty

**Affiliations:** 1 Population Health Sciences Institute, Faculty of Medical Sciences, Newcastle University, Newcastle upon Tyne, UK; 2 National Institute for Health and Care Research (NIHR) Applied Research Collaboration North East and North Cumbria, Newcastle, UK; 3 National Institute for Health and Care Research (NIHR) Innovation Observatory, Newcastle University, Newcastle upon Tyne, UK; 4 Evidence Synthesis Group, Population Health Sciences Institute, Faculty of Medical Sciences, Newcastle University, Newcastle, UK

**Keywords:** Long-term care facilities, Deterioration tools, Acute illness

## Abstract

**Background:**

Acute deterioration describes a rapid decline in health due to short-duration illnesses. This is an important topic for older adults living in long-term care facilities (LTCF). Signs of acute deterioration are often subtle, and there is no standardised system to manage it. The aim of this review is to scope the range of deterioration tools used in LTCFs, and to describe how they have been evaluated.

**Methods:**

A scoping review was conducted in accordance with the Joanna Briggs Institute methodology. Searches of five (MEDLINE, APA PsycInfo, Embase, CINAHL, HMIC) electronic databases (2013–2023, updated 2025) and relevant websites were followed by title/abstract (by two authors independently) and full-text screening. Eligible studies involved tools used to manage acute deterioration for adults > 65 years in LTCFs. Experimental and observational study designs were eligible, including quality improvement projects. No country or language restrictions were imposed. A narrative synthesis was conducted.

**Results:**

Twenty-six studies were included (23 peer-reviewed articles, two conference abstracts, one dissertation) after screening 5958 articles. A majority were from the UK (*n* = 10) and USA (*n* = 9), with small numbers from other high-income countries ((Australia (*n* = 2), Canada (*n* = 2), Sweden (*n* = 2), Switzerland (*n* = 1)). Studies employed a wide range of methodologies, with only one randomised study, and tools were frequently evaluated as part of multi-faceted interventions. The majority of studies described an intervention in which SBAR (situation-background-action-recommendation) (*n* = 15), National Early Warning Scores (*n* = 7) or STOP AND WATCH (*n* = 4) were a component. Studies used quantitative (*n* = 21) and qualitative (*n* = 9) methods to evaluate tools. Outcome measures were heterogeneous, with no data on resident experience. The majority of studies concluded potential benefit from using deterioration tools. There is some evidence that LTCF staff perceive tools, especially SBAR, as improving confidence in managing acute deterioration and aiding communication with external healthcare professionals.

**Conclusion:**

Despite policy drivers to use deterioration tools in LTCFs, there is no robust evidence to support this. Direct benefits for resident care have not been demonstrated. Further research is required to compare tools to standard care, measure the impact on resident experience, and to determine if deterioration tools should become part of routine care in LTCFs.

**Supplementary Information:**

The online version contains supplementary material available at 10.1186/s12913-025-12534-x.

## Background

Residents living in long-term care facilities (LTCF) represent a large and important population in our society. Approximately 420,000 people live in care homes in the United Kingdom (UK) [1], representing a bed base three times that of the hospital sector [2]. LTCFs are institutional settings where older adults live and receive long-term social and health care. Older adults in LTCFs are living with complex care needs, high levels of frailty, dependence and complex multimorbidity [3–5]. LTCF residents are susceptible to severe forms of acute illness. This is a term used to describe illnesses of short duration, either an exacerbation of a pre-existing problem, or the rapid onset of a new condition [6], for example infections like pneumonia. Acute illnesses can cause rapid deteriorations in the health of residents living in LTCFs, and they experience high rates of emergency hospital attendance and admission [7]. A significant proportion of emergency admissions to hospital may be avoidable [8–10]. Improving how LTCF and healthcare staff work together to respond to acute deterioration is a key component of enhancing care for residents living in LTCFs, as it may signal illness requiring active treatment in the LTCF or in hospital, or a need to consider end-of-life care.

Acute deterioration poses a challenge for both LTCF staff, and healthcare professionals external to the care home. External healthcare professionals who respond to concerns from LTCF staff depend on the specific healthcare setting, but may include general practitioners, specialist doctors (with training in geriatrics), or specialist nurses [11]. The main challenges for LTCF and health staff are that signs of acute deterioration are often subtle or absent [12], and deterioration trajectories are frequently unpredictable [13]. Older adults have a tendency to under-report symptoms to their caregivers [14]. Therefore, LTCF staff often rely on informal observations (e.g. reduced mobility), and their intuition, to alert them to an evolving illness that could deteriorate rapidly. Objective indicators of acute deterioration, such as vital sign measurement, may also contribute to the initial assessment, especially when on-site nurses are part of the LTCF care team.

When LTCF staff are concerned, they are required to decide how and when to communicate concerns within the LTCF, or whether to escalate their concerns to external healthcare professionals. When escalation occurs, healthcare staff frequently receive information from LTCFs that lacks objective data on which to base treatment decisions. This complicates the next stage in the response to acute deterioration, during which healthcare professionals work with care home staff and residents (or their friends/family) to formulate a care plan that is consistent with resident wishes. The stages in the response to acute deterioration described above are shown in Fig. 2.

There is not a standardised system for LTCF and healthcare staff to identify and respond to acutely deteriorating residents [15]. Therefore, policymakers are widely implementing interventions/tools that aim to improve the management of acute deterioration but without evidence on how they impact resident care and the experiences of staff working in LTCFs. There is a strong policy incentive for deterioration tools to be implemented in LTCFs, such as the National Early Warning Score (NEWS) in the UK [16]. Up-scaling of remote monitoring in LTFCs and the implementation of interventions to enhance the response to deterioration have also been accelerated by the COVID-19 pandemic [17].

The evidence base about acute deterioration in LTCFs is evolving. Hodge et al. conducted a scoping review to describe how acute deterioration is identified by care home teams [18]. They concluded that this is a complex process, ‘context sensitive,’ also depending on factors external to care homes. However, deterioration tools were not the subject of this review. Subsequently, Daltrey et al. conducted a review of evidence from peer-reviewed journals about models of care and ‘clinical patterns of deterioration’ in residential aged care facilities [15]. This included some coverage of deterioration tools to support registered nurses in responding to deterioration. However, no previous reviews have focused solely on the role of deterioration tools or how they have been evaluated, despite the policy drivers to increase the use of tools. Our review builds on the current evidence base by focusing specifically on deterioration tools. The aim is to scope the current evidence base about which deterioration tools are being used in LTCFs and what outcome measures have been used in their evaluation, as well as the implementation challenges. Consequently, this review aims to extend the evidence base identifying promising tools for future development, and proposing outcome measures necessary to evaluate their impact on resident care and effectiveness in future work.

### Aim

The aim of this review is to scope the range of deterioration tools employed in LTCFs, and to describe how they have been evaluated in this setting.

#### Objectives

Evidence will be synthesised on the current tools used in managing LTCF resident acute deterioration:

1. To identify the range of tools employed to identify and manage resident acute deterioration in LTCFs.
2. To describe the outcomes used to evaluate deterioration tools in LTCFs.
3. To determine if existing evidence suggests that one tool(s), or particular component(s) of composite tools, should be preferred over any other(s) for future development.

## Methods

A scoping review was conducted as the body of evidence for deterioration tools in LTCFs was expected to be complex and heterogenous [19]. This review was conducted in accordance with the Joanna Briggs Institute (JBI) scoping review methodology [19].

### Search methods

The search strategy was developed in MEDLINE (OVID) and combined MeSH terms, keywords, and synonyms for the type of facility, such as ‘long-term care facility,’ ‘nursing home’, and ‘care home’, with a set of terms related to specific tools, for example ‘Early Warning Score’, ‘Stop and Watch’, and ‘SBAR’ as well as more generic terms for deterioration tools. Searches were translated to APA PsycInfo (OVID), Embase (OVID), and HMIC (Health Management Information Consortium) and CINAHL (EBSCOhost). Searches were restricted to 2013 to ‘current’ where the database allowed and carried out on the 4th of April 2023. The searches were updated on the 7th of January 2025, starting from January 2023 (which may mean that there is a small degree of overlap with the initial search). This search strategy aimed to capture the majority of evidence which has emerged in the last decade. The records from each database searched were imported into EndNote 21 where they were combined and deduplicated. Specific websites were scrutinised, including The Health Foundation, The King’s Fund, the British Geriatrics Society, the Nuffield Trust, and the International Long-Term Care Policy Network. Consultation with the academic and professional networks of the study team was undertaken, including targeted emails to academics and clinicians working in this field. Full details of the searches undertaken along with the search strategies are given in the Supplementary materials.

#### Inclusion and exclusion criteria

##### Participants

Eligible studies focused on older adults (> 65 years) living in LTCFs. Residents living in facilities with registered nurses on-site (for example nursing homes), and those without were eligible for inclusion.

##### Intervention

For the purpose of this review, the intervention was defined as a tool used by LTCF staff and/or healthcare professionals to identify and/or manage residents who may be experiencing a deterioration in health due to an acute (short-duration) illness [6], in the LTCF setting. There is no specific definition for acute illness/deterioration in the setting of LTCFs.

To be eligible, studies were required to, (a) describe a deterioration tool being used in the delivery of resident care in LTCFs (studies reporting on training on tools only were excluded), and (b) measure outcome(s) that relate specifically to deterioration tool use in LTCFs. Models of care and multi-modal interventions that incorporated deterioration tools but did not report outcomes specific to the tool itself were excluded.

##### Study designs

Experimental and observational study designs were eligible for inclusion, including quality improvement projects and service evaluations. Unlike previous reviews, eligible sources of evidence were not limited to papers published in peer-reviewed journals, as the authorship team were aware that evidence about deterioration tools in LTCFs is also likely to be found in reports and service evaluations. Conference abstracts were included if they contained sufficient data to describe an intervention and measured outcomes. There was no restriction in terms of publication country or language. Previously published reviews were not eligible for inclusion but bibliography searches of these studies were conducted.

##### Exclusions

Younger adults (< 65 years-old) living in LTCFs and Deterioration tools used in hospitalised residents of LTCFs were excluded. Studies not reporting outcomes to evaluate tool use (for example a description of a tool only) or studies in which tools had not been used to influence resident care (for example deterioration tools used retrospectively to assess quality) were excluded. Outcomes that assessed staff knowledge following training (even if focused on a deterioration tool) were excluded.

#### Study selection

The selection process consisted of two levels of screening, (a) the title and abstract, and (b) full-text papers. For the first level of screening, the titles and abstracts of all retrieved studies were screened against the inclusion criteria by two reviewers. Rayyan, an online screening tool [20], was used for title and abstract screening, with the author blinding function enabled. Any disagreements were discussed between the two authors, or with input from a third author, to achieve a consensus. In the second step, the full text was assessed against the inclusion/exclusion criteria by the primary author and all selections were checked by a second author to ensure consistency.

#### Data extraction

Data were extracted from included studies into a Microsoft Excel spreadsheet, which had been jointly developed by two authors. Data extraction was performed by the primary reviewer and checked by a second author. Data on the following variables were extracted:

1. Study information- author, year of publication, type of publication.
2. Study design – as stated by study authors.
3. Study setting - country of study origin, type of LTCF.
4. Description and type of deterioration tool (and co-interventions if relevant).
5. Outcome measures relating to deterioration tool use.
6. Key findings/conclusions.

#### Data synthesis

A descriptive summary with data tables was produced to summarise the literature using a narrative synthesis, as the data were not suitable for pooling. Data were grouped according to the deterioration tool type/point at which they are intended to act on the deterioration response pathway.

## Results

**Fig. 1:**
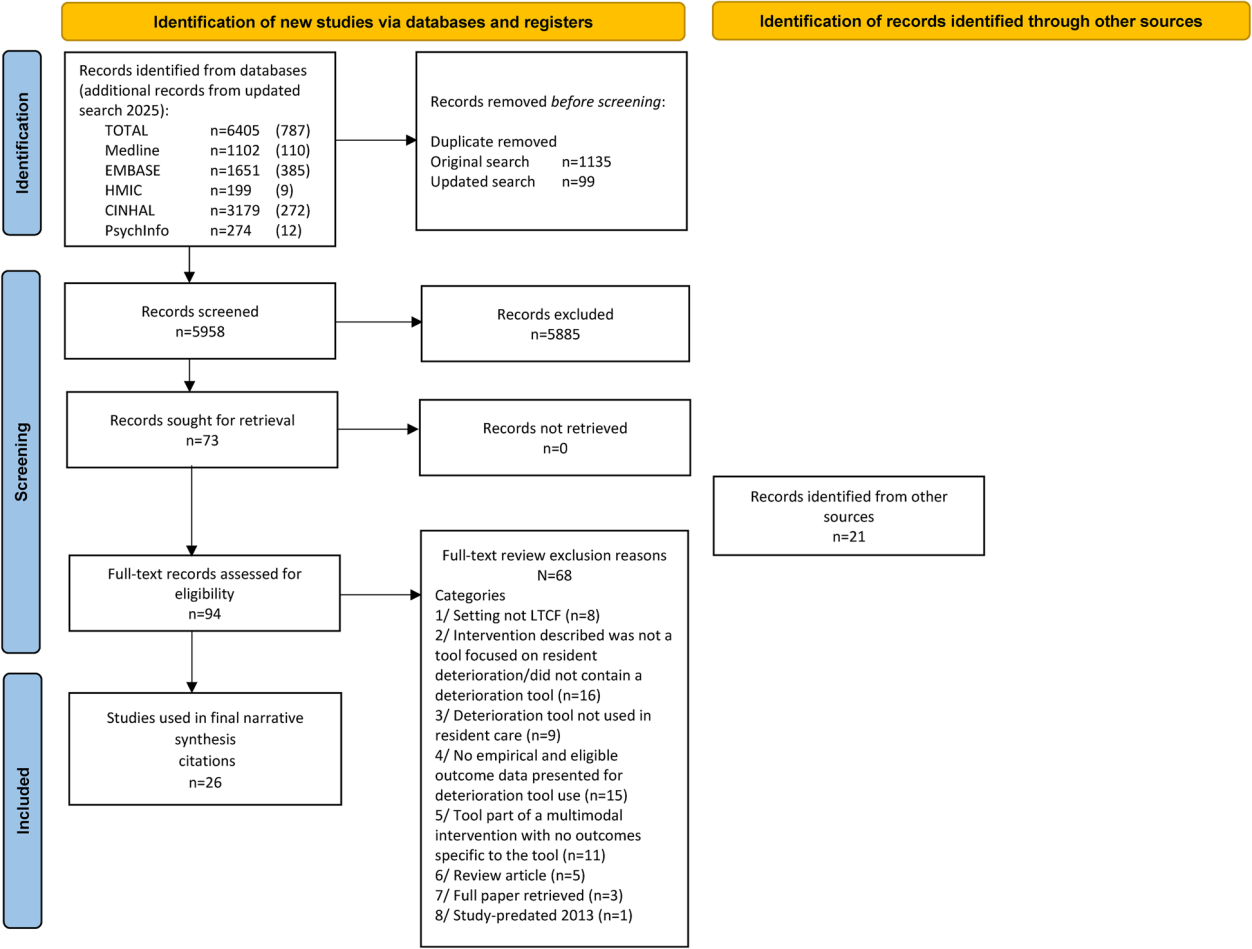
PRISMA diagram showing study selection (adapted from PRISMA diagram - https://www.prisma-statement.org/prisma-2020-flow-diagram)

A total of 5958 titles and abstracts were screened from database searches, resulting in full-text screening of 73 studies from database searches. Full text review was also conducted for 21 studies retrieved through hand/bibliography searching. The reasons for exclusion following full-text screening are displayed in the PRISMA diagram (Fig. 1). The commonest exclusion category was tools/interventions with an alternative focus to resident deterioration (*n* = 16), followed by studies of deterioration tools with no eligible, empirical outcome data specific to the tool itself (*n* = 15). Authors were contacted on four occasions to aid decisions about eligibility. Overall, 26 studies were eligible for inclusion, twenty [17, 21–39] from database searches and six from hand/bibliography searching [38, 40–44]. A total of 23 studies were from peer-reviewed journals [17, 21, 22, 24–32, 35–45], with two conference abstracts [33, 34] and one dissertation [23]. Figure 1 shows the study selection process.

### Study setting and design

The majority of studies (*n* = 20) presented data on the impact of tool use [17, 21–24, 27–34, 36, 39–41, 43–45], with others addressing tool validation [25, 35, 37, 38, 42]. None of the identified studies presented outcomes for deterioration tool use compared to standard care (with no deterioration tool use). One study compared a two different deterioration tools (a customised version of SBAR with standard SBAR) within different LTCFs [30]. There was a high degree of heterogeneity in study design. One was a randomised study but there was insufficient intervention uptake to assess effectiveness [45]. A significant number of studies (*n* = 10) of included studies were conducted at a single-site LTCF [23, 28, 29, 31, 32, 34, 36, 37, 41, 43].

A majority of studies were conducted in the UK (*n* = 10) [17, 21, 22, 25, 26, 28, 35, 39, 44, 45] or the USA (*n* = 9) [23, 29–34, 36, 40], with other studies from LTCFS in Australia (*n* = 2) [24, 41], Canada (*n* = 2) [37, 43], Sweden (*n* = 2) [38, 42], and Switzerland (*n* = 1) [27].

### Deterioration tools used in LTCFs

Characteristics and key findings of included studies are described in Table 1 and summarised in Table 2. Most studies described an intervention in which the SBAR (situation-background-action-recommendation) tool [17, 22, 23, 27, 29, 31, 32, 34, 36, 40, 41, 44, 45] or a tool based on SBAR [24, 30] (*n* = 15) was a component. The National early Warning Score (NEWS), a ‘track and trigger’ systems using vital sign measurement, was a component of interventions in seven studies based in the UK [17, 21, 22, 25, 26, 39, 44]. The STOP AND WATCH tool was a component in four studies [27, 29, 33, 45], delirium screening tools [35, 37] and the Early Detection of Infection Scale (EDIS) [38, 42] the subject in two papers, Practical Routine Elder Variants Indicate Early Warning for Emergency Department (PREVIEW-ED) [43] and Significant Seven [28] in one study.

**Table 1:** Study characteristics, outcome measures and findings

**Author, publication** **year**	**Publication type**	**Country**	**LTCF type**	**Study type/ design– author reported ** **(deduced if not stated)**	**Intervention, co-interventions**	**Outcomes reported**	**Key findings reported relevant to resident care**	**Author conclusions**
O’Neill 2017 [41]	Peer-reviewed journal	Australia	Nursing home (*n*=1 with 94 beds))	(Qualitive service evaluation)	Residential Acute Deterioration Detection Index (RADD) (traffic light system) and SBAR Co-intervention– deterioration tools were part of a broader hospital avoidance programme, including other decision-support tools such as clinical management guidelines. Other facets to the intervention included advanced clinical skills training, specialist clinical support and medical equipment.	Qualitative– focus groups with LTCF staff. Outcomes were specific to the deterioration tools.	It was not clear how often SBAR was used, but its benefits were acknowledged, specifically that it helps to give a structure for communicating concern about deterioration that is ‘to the point.’ Benefits reported of using SBAR and the traffic light system to increase LTCF staff confidence, for example ‘I think that traffic light thing does give you the confidence to deal with it, even if the RN can’t get down there you know you’re only talking on the phone explaining, doing the SBAR thing so you’re basically following that until it gets to the stage where you definitely need some more help, so I think that’s a critical part I like.’	LTCF welcome the hospital avoidance programme, including reporting benefit from the deterioration tools. However, staffing and a ‘shift in workload’ are potential barriers**.**
Russell 2020 [22]	Peer-reviewed journal	UK	Care homes (*n*=47)	‘Service evaluation’ (qualitative evaluation)	NEWS	Qualitative interviews (*n*=21)– care home staff, healthcare professionals, healthcare manager	Themes from qualitative interviews: 1/ The benefits of NEWS, including ‘backup’ of care home staff judgements. 2/ ‘Inhibitors to engagement’ (1/3 of care homes used NEWS regularly), including the variation on skills and expertise within different care homes. 3/ ‘Shortfalls in communication,’ and deficiencies in training and implementation and support. Mapping to Normalisation Process Theory constructs, the authors had identified ‘may real world barrier in its (NEWS) implementation’ according to these constructs.	NEWS ‘could enhance’ the care of acutely deteriorating residents, improve staff (care home) confidence and improve communication. The implementation strategy did not account for the complexity of the intervention. Challenges to engagement including ‘competing priorities’ (for care home staff) and training. The appropriateness of NEWS in care homes requires further study and implementation requires greater involvement from care home staff and primary care services.
Sampson 2020 [45]	Peer-reviewed journal	UK	Nursing homes (*n*=14, 7 control, 7 intervention)	‘Pilot cluster randomised controlled trial’	Stop and Watch SBAR Co-intervention– care pathways– a 2 step clinical guidance and decision-support system. The pilot ran for 10 months.	The study aimed to collect data on multiple domains including unplanned hospital admission, resident quality of life (using EQ-5D-5L), and qualitative data (to understand barriers and facilitators to implementation).	None of he of the nursing homes implementation tools as planned. Across the 5 participating LTCFs, only 16 STOP and Watch forms were completed (11 from one single site)– median of one per month. It was challenging to collect data on hospital admissions (as well as ambulance service and primary care contacts).	Successful recruitment and retention of residents, staff and family carers. There was limited engagement with the intervention tools. No LTCF implemented the tools as expected. There was no evidence of harm. This particular complex intervention does not warrant a future definitive trial as it was not implemented.
Stocker 2021 [17]	Peer-reviewed journal	UK	Care homes (*n*=7, approximately 280 beds)	‘Service evaluation’ (qualitative evaluation)	NEWS Co-intervention: education and support, enhanced features e.g. clinical picture taking	Qualitative– care home (*n*=10) and health service staff across 7 care homes.	The intervention was thought to have enhanced the response to acute deterioration in care homes during the COVID-19 pandemic. Healthcare professionals saw the benefit of NEWS assisting in remote monitoring and clinical assessment. Care home staff felt that NEWS supported and ‘empowered’ the decisions they were making about acutely unwell residents, provided a ‘common language,’ and acted as an ‘adjunct to staff intuition.’ Themes from the qualitative interviews were: >> ‘NEWS intervention during the pandemic: an adjunct to COVID-19 identification.’ >> ‘Use of individual physiological observations to identify COVID-19.’ >> ‘Using the NEWS intervention to remotely monitor health.’ >> ‘Empowerment of care home staff with the National Health Service agenda and language.’ >> ‘Centrality of training relationships and clinical support for accelerated implementation of NEWS during a pandemic.’ >> ‘Training and implementing the NEWS intervention in care homes during the pandemic.’ >> ‘Ongoing clinical support for care homes during the pandemic.’	NEWS (and associated education and support package) had a useful role during the COVID-19 pandemic, enhancing remote clinical assessment and offering care home staff reassurance and ‘structure in their role.’
Porter 2021 [29]	Peer-reviewed journal	USA	Skilled-nursing facility (*n*=1, 45 beds)	‘Quality improvement, one-group pretest-post-test’	1/ Sepsis screening tool (uses the SIRS criteria) 2/ Stop and Watch 3/ SBAR Co-intervention– sepsis education.	Quantitative– sepsis screening frequency Outcomes related to staff knowledge changes pre and post education programme were not eligible for inclusion	2068 sepsis screens were conducted over a 3 month period (*n*=30 not completed/lacked information). Over a 3-month period, 2,068 sepsis screens were performed and 1.5% (*n* = 30) of screens were either not completed or lacked sufficient documentation. Amongst the 2,038 sepsis screens, 0.2% (*n* = 4) screened positive for sepsis. ‘Three (75%) of four positive screens for sepsis resulted in timely notification of the charge nurse and physician followed by transfer to an acute care hospital.’ (timely communication measured by charge nurse notification within one hour).	There is value in providing staff with a sepsis screening tool (SIRS criteria), as well as cognitive changes with STOP AND WATCH (and value in providing sepsis education). SBAR supports staff’s findings that could indicate sepsis. The authors assert that sepsis screens resulted in timely resident treatment and avoided hospitalisations.
Montgomery 2023 [24]	Peer-reviewed journal	Australia	Residential Aged Care Facilities (RACF) (*n*=8)	‘Quasi-experimental pre-post design’ (before-after study)	1/ RACF Emergency Decision Index (REDI) 2/ Clinical Handover Assessment Tool (CHAT), based on SBAR.	Survey data before (T0) and after (T1, 6 months post- implementation)– self-confidence RACF staff in the management of acutely unwell residents	254/284 (90%) responded at T0, 20% (51 respondents) response rate at T1. Significant increase in confidence to assess and manage acutely unwell residents following implementation of the REDI and CHAT (*p* = 0.003 and *p* = 0.006, respectively) tools. Baseline Confidence in Assessment Scale and Confidence in Management Scale scores differed significantly post-implementation of the REDI and CHAT tools (*p* < 0.001). Improvement was shown across all communication domains. The greatest improvement in confidence was in communicating with the hospital outreach service from mean 3.37 (SD = 0.94) to mean 3.86 (SD = 0.61) (max. score 5). Participants reported ‘sometimes’ using the REDI and CHAT tools 52% and 44% respectively.	Both tools increased confidence of RACF in assessing and managing acute deterioration, and communicating about acute illness. The tools could increase staff competence. The authors state that they ‘cannot infer that self-efficacy leads to improved performance.’
Basinska 2022 [27]	Peer-reviewed journal	Switzerland	Nursing homes (*n*=11)	‘Convergent mixed-method design within a hybrid type-2 effectiveness-implementation study.’ (mixed-methods service evaluation)	1/ ISBAR (Introduction, Situation, Background, Assessment, Recommendation) 2/ Stop&Watch Co-intervention: Third intervention element - nurses providing on-site geriatric support (INTERCARE nurse)	Quantitative– uptake, acceptability (Acceptability of Intervention Measure, AIM) and feasibility (Feasibility of Intervention Measure, FIM) Qualitative– 22 focus groups with care home staff– tools and implementation.	1/ Acceptability/feasibility measures from 573 care workers from 11 nursing homes. STOP&Watch Acceptability 68% (registered nurses, licensed practical nurses, nurse aides) Feasibility 79% (registered nurses, licensed practical nurses, nurse aides) Uptake 78% (nurse aides) ISBAR Acceptability 74% Feasibility 85% Uptake 77% 2/ Qualitative results (108 care workers, 22 focus groups) specific to ISBAR and STOP&Watch ISBAR– RNs and LPNs found the instrument understandable and requiring few changes to their normal routine. They perceived it be straightforward to use when evaluating resident and communicating with healthcare professionals. RNs and LPNs described increased confidence during conversations with healthcare professionals, ‘leading to well-informed, timely decisions’ for resident care. STOP&Watch– nurse aids found it hard to use and difficult to understand, and some forgot to take with them when documenting resident observations. Nurse aids initial resistance to using STOP&Watch changed as they became more aware of the changes they needed to look for, and saw that these were being taken seriously. STOP&Watch and ISBAR– daily practice was adapted to facilitate use. Different settings mean that care teams needed to work out how to use these deterioration tools, and they appreciated adaptations that responded to their views. There was initial scepticism about the tools’ relevance and potential to increase workload. In some cases, scepticism lessened but those who didn’t recognise the practical value didn’t use the tools.	Considerable variations in the acceptability, feasibility and uptake of interventions. Qualitative findings ‘highlight differences in the nursing homes ’ internal implementation processes,’ and various ‘individual barriers and facilitators to implementation.’ ISBAR (and INTERCARE nurse role) consideredacceptable and feasible, taken up by > 70% of care workers. Lower levels of acceptability, feasibility and uptake with STOP&Watch compared to ISBAR (and INTERCARE nurse).
Nwolise 2024 [44]	Peer-reviewed journal	UK	Care homes (*n*=35)	‘Mixed-methods approach, semi-structured interviews and online survey.’	RESTORE2 is a composite deterioration, including 1/ ‘soft signs,’ 2/ NEWS, 3/ SBARD	Quantitative– online survey (20 respondents) Qualitative– semi-structured interviews (*n*=15)	35 care home staff participated. Findings were grouped according to three main themes: 1) RESTORE2 training and use > Implementation of RESTORE2 was low; components of RESTORE2 were often used as opposed to all elements. > RESTORE2 use was mainly by nurses. > 3/20 survey respondents had never heard of RESTORE2; 5/20 had heard of RESTORE2 but never used it; 2/20 stopped using RESTORE2; 2/20 used RESTORE2 occasionally, 8/20 regularly. > Training roll-out was ‘slow and patchy,’ but most staff found it ‘helpful’ and were satisfied with its delivery. 2) Benefits of RESTORE2 > From the qualitative interviews, RESTORE2 was perceived to have benefited residents and their family/friends, through prompt responses to acute deterioration. Views were mixed about the impact on healthcare utilisation. > Lack of agreement with survey data about the impact of RESTORE2 on care provision and the management of deterioration. However, there was some agreement that RESTORE2 improved staff confidence. 3) Implementation challenges and ‘moving forwards.’ > Multiple implementation challenges highlighted e.g the belief that RESTORE2 was ‘medically oriented’ and more suitable for use in nursing homes than residential homes; perception that residential homes lack training and sufficient staff levels to measure NEWS2. > Residential care staff perceived the use of NESWS2 to be ‘complicated.’ Some survey respondents described SBARD as ‘time-consuming’ and RESTORE2 as ‘lengthy.’	RESTORE2 has potential benefits e.g. improved confidence to identify acute deterioration and to escalate/communicate concerns. The authors state that RESTORE2 has the potential to reduce healthcare use, such as hospital admissions. The ‘myriad of challenges that affect use and successful implementation’ need to be considered. Further quantitative and qualitative research (including exploration of the resident perspective) is required.
Renz 2013 [31]	Peer-reviewed journal	USA	Skilled-nursing facility (*n*=1)	‘Repeated measure, quality improvement’	(INTERACT II) SBAR	Questionnaire (pre and post) administered to care home and healthcare staff (with free text comments )– 1) nurse satisfaction with nurse-medical provider communication 2) medical provider perception of nurse/medical provider communication 3) adherence to SBAR.	Nurse satisfaction with nurse-medical provider communication demonstrated improvement (not statistically significant) in the majority of questionnaire items. 87.5% of nurses (*n*=28) found SBAR helpful. 69% (*n*=22) found no limitations but 28% (*n*=9) found SBAR to be time-consuming. 5 out of 7 healthcare professionals reported that the quality of communication with LTCF staff (nurses) had improved post-SBAR implementation. 6 reported that nurses were consistently providing adequate information about residents who were deteriorating and that the information influences decision making about hospital transfer. 65 SBARs completed over a 3 month period. 78% (*n*=51) complete documentation, 22% (*n*=14) had missing documentation. SBAR was completed in a timely manner and only one resident change of condition lacked SBAR completion (98% compliance).	Implementation of SBAR ‘suggests improvement in nurse satisfaction with communication.’ Study findings support the use of SBAR to help address the issues of complete documentation and time constraints.
Renz 2015 [32]	Peer-reviewed journal	USA	Skilled-nursing facility (*n*=1)	‘Single-site repeated measures design, quality improvement’	SBAR	Quantitative– rate of hospital transfer, cost-benefit analysis.	SBARs completed for all unplanned hospital transfers - timeliness of the SBARs was 72% during the first month. In the 2^nd^ to 3^rd^ months, 90% completed in a timely manner, and 100% completion was sustained. The authors present a table entitled ‘effect of SBAR implementation on hospital transfers,’ and state that this shows that the ‘total number of hospital transfers declined over the ‘early period of SBAR implementation.’ An overall reduction in avoidable hospital transfers (*n*=10) over a 4 month period. Avoidable hospital transfers remained constant across the 4 months of implementation (mean = 1.75 transfers per month). The number of 30-day hospital readmissions steadily declined over 4 months. Total project cost = $4,355.00 (including material costs, staff replacement, data analysis, and medical record review time) Potential cost savings (resulting from an overall reduction in avoidable hospital transfers (*n*=10) over a four month period) = $109,350 (based on an average cost of $10,935 for hospital admissions from nursing homes).	SBAR was completed thoroughly with timeliness and ‘high-quality clinical data.’ SBAR is a ‘feasible method to provide structured communication between nurses and medical providers.’ There is the potential for SBAR to positively influence decision-making to hospitalise residents. The interdisciplinary project ‘can be implemented at low cost in any long-term facility.’
Jarboe 2015 [23]	Dissertation	USA	Skilled-nursing facility (*n*=1)	‘Before and after’	(INTERACT) SBAR (Situation– Background– Action -Recommendation)	Quantitative– unplanned hospital transfer	Pre and post-SBAR implementation, there was no significant difference in overall resident transfers (*p*=0.482). There was no significant difference in the preventable transfers (*p*=0.927),'discretionary care' (*p*=0.547),'emergent' (*p*=0.565),'admitted to hospital' (*p*=0.662),'not admitted' (*p*=0.468) groups. However, there was a significant difference'futile care' group (*p*=0.041).	No significant decrease in unplanned hospital transfer.
Ashcraft 2017 [30]	Peer-reviewed journal	USA	Nursing homes (*n* =2, 1 control + 1 intervention)	‘Pre-post, quasi-experimental’ (with a control and intervention nursing home)	Standard SBAR Customized SBARc using a ‘formal process and guided by Sensemaking.’	Quantitative– nurse-clinician communication, resident transfer Qualitative– Two focus groups (*n*=5 control, *n*=7 in intervention)	Quantitative - Significant difference in the use of the SBARs versus the SBARc tool. - More frequent use of SBAR tools in the control site (×^2^= 42, *p* < 0.0001). - Completion of SBAR (used as a measure of quality of communication) was greater at the control site (t =−0.50, *p*= 0.62). - 8.55% of reported communication events at the experimental site were recorded using SBARc, compared to 43% with SBAR at the control site. - Transfer rates between experimental and control sites similar (SBARc-7.53% versus SBARs-6.85%; X2 = 0.04, *p* = 0.84). Qualitative SBAR was not used consistently at either facility. Staff saw it as ‘an expectation of management as well as institutional policy.’ Reasons for non-use included, a) use of an alternate documentation location (e.g. transfer documents, ‘(b) faxing of notes to clinicians, (c) placement of notes on the chart, (d) phone calls already documented in physician orders, and (e) use of SBAR to document only acute change in resident status.’ SBAR was perceived to help when resident deterioration meant they may require hospital transfer. When this happened, nurses used the SBAR form to directly record features of deterioration. Nurses felt they ‘thought about problems in the SBAR format and did not need a form.’	The SBAR format did not influence nursing communication with healthcare professionals. SBAR customisation did not improve usage. SBAR usage alone was not significantly related to hospital transfer. There was a ‘weak but significantly positive relationship’ between completion of the recommendation or request sections of SBAR and the risk of resident hospital transfer. Communication about residents was ‘best captured using multiple approaches, including SBAR, Prover Logs, and face-to-face meetings.’
Devereaux 2016 [34]	Conference abstract	USA	Skilled nursing/post-acute care facility (*n*=1, 139-bed)	‘Quasi-experimental one group pre/post- Test’ (before-after study)	Condition specific SBAR	Quantitative– 3 month pre/post implementation– total transfers, unplanned hospital admissions and 30-day readmissions.	3 months after implementation, it was observed that there was a significant reduction in rates of 1) total hospital transfers (0.44 vs. 0.24, *p*<.001), 2) all hospital unplanned admissions (0.36 vs.0.18, *p*-value unclear from the manuscript), 3) 30-day readmissions (0.12 vs. 0.04, *p*=0.012), 4) avoidable hospital transfers (0.26 vs. 0.09, *p*<0.001), 5) avoidable hospital admissions (0.15 vs. 0.05, *p*=0.007), 6) transfers due to pneumonia (0.52 vs. 0.17, *p*=0.018).	Using condition-specific SBARs as a deterioration tool reduced unplanned hospital transfers, hospitalisations, and 30-day readmissions. When these occurred, they were more likely to be unavoidable. Limitations of small sample and single facility study.
Owen 2019 [36]	Peer-reviewed journal	USA	Nursing home (*n*=1)	‘Exploratory sequential mixed method with a pre/post quasi-experiment’	SBAR	Qualitative– Nurse and physicians	Six aspects of the communication process: (a) Nursing Knowledge and Information Presentation; (b) Focused Communication; (c) Sustaining Conversation; (d) Shared Meaning; (e) Event Resolution; and (f) Documentation Nurses perceived that SBAR helped communication with physicians, helping nurses to organise their thoughts prior to calling physicians and to provide the necessary information.	‘Shared meaning and training in SBAR use as a means of communication (versus documentation) has the potential to provide for the development of stronger interventions with structured communication.’
Luna 2023 [40]	Peer-reviewed journal	USA	Nursing homes (*n*=2)	‘Qualitative, descriptive study’	SBAR was a theme that emerged in this qualitative study that aimed to ‘assess the different features of interprofessional nursing home relationships and communication between nurses and providers (e.g. physicians and nurse practitioners) to understand how they could impact unnecessary rehospitalisation decisions.’	Qualitative– two focus groups.	SBAR was one of the 5 main themes In the theme of ‘interprofessional communication,’ nurses were aware of SBAR but ’did not always reference it explicitly.’ They viewed it as a routine tool for assessment of residents and communicating with healthcare providers external to the LTCF. They used SBAR when there was a change in a resident’s condition. When providers and nurses use SBAR, there is increased shared understanding. Providers were less familiar with SBAR, emphasising vital signs, clinical examination and knowledge of the individual resident. They listened to the SBAR components, alongside and changes in resident condition from baseline reported by nurses.	SBAR was seen as facilitating ‘timely and accurate communication,’ but some nurses ‘stated that they did not feel welcome to engage in the problem-solving process.’ Trust and mutual respect enhanced the impact of tools like SBAR. SBAR is a way to facilitate communication between nurses and ‘providers,’ but it is not used consistently. Nurses may informally follow the components of SBAR, without using the exact structure.
Barker 2019 [25]	Peer-reviewed journal	UK	Care homes (*n*=46)	‘Cross-sectional study’	NEWS	Quantitative– Comparison of NEWS measured as a baseline measure and when carer concern.	A total of 19,604 NEWS analysed from 2,424 residents. Overall median NEWS = 2 (interquartile range (IQR) 3 and range 0–13) Median NEWS at baseline = 1 (IQR2 and range 0–12) Median NEWS if carer concern = 2 (IQR 4 and range 0–12). The proportion of low NEWS (0-2): 76% for routine measurements, 62% for carer concern. Intermediate category scores (NEWS 3–4): 18% for routine measures, 21% in the staff concern group. Overall, only 9% of NEWS measurements were in the high range (NEWS 5 or 6) and 4% critically high (NEWS ≥7). High and critically NEWS were more common in the staff concern group (11% high, 6% critically high), compared with the routine group (5% high, 2% critically high).	Measurement of NEWS in care homes appears to be feasible, and the majority of scores were not elevated at baseline. Scores were higher when there was carer concern (about a change in resident health status). The distribution of NEWS measurements was with other settings.
Stow 2021 [26]	Peer-reviewed journal	UK	Care homes (it was not possible to ascertain care home numbers– see key findings column)	‘Ecological time-series study’	NEWS	Quantitative– Association between NEWS, and its components, and COVID-19 deaths	29 656 NEWS recordings across 46 local authority areas, 480 unique care home identifiers (representing an individual care home or a ‘distinct unit’ within a care home) 6,464 care homes residents with at least one NEWS. Between 23^rd^ of March 2020 and 10^th of^ May 2020: 5753 deaths (1532 involving COVID-19 and 4221 other causes). A rise in the proportion of above-baseline NEWS was observed for week beginning 16^th^ of March 2020. This was ‘followed 2 weeks later by an increase in registered deaths (cross-correlation of *r*=0.82, *p*<0.05 for a 2 week lag) in corresponding local authorities.’ ‘The proportion of above-baseline oxygen saturation, respiratory rate and temperature measurements also increased approximately 2 weeks before peaks in deaths.’	The authors stated that NEWS could ‘contribute to COVID-19 disease surveillance’ and asserted that ‘oxygen saturation, respiratory rate and temperature could be prioritised as they appear to signal rise in mortality almost as well as NEWS.’
Hodgson 2022 [21]	Peer-reviewed journal	UK	Care homes (*n*=4)	‘Two-strand convergent parallel design’ (mixed-methods)	National Early Warning Score (NEWS) digitally recorded on a Bluetooth device. Co-intervention: the digital device also recorded Barthel Index for Activities of Daily Living, and Rockwood frailty score.	Quantitative (276 residents, 4 care homes), admission rate. Qualitative– Care home staff experience of using NEWS.	Baseline NEWS was recorded. Following subsequent measures, 66.2% (*n* =233) of residents were ‘referred to another service,’ 73.4% (*n*=174) to their own GP, 81.0% (*n*=192). 9.91% (*n*=31) NEWS readings for 26 residents led to hospital admission (22 residents admitted hospital once, 3 residents twice, 1 resident three times) A ‘statistically significant link between NEWS score and hospital admissions (chi-square=0.573, *p*<0.0001, Cramer's V=0.405)’ was reported. Themes from qualitative interviews– NEWS as: 1/ measurements as “proof” 2/ efficient diagnosis 3/ empowering communication 4/ empowering role 5/ decision-making processes	NEWS benefits the care provided, has the ability to highlight the need for hospital admission, improves communication, and empowers staff.
Siriwardena 2024 [39]	Peer-reviewed journal	UK	Care homes (number not stated)	Retrospective cross-sectional study	NEWS2 (measured by ambulance staff)	Quantitative– emergency conveyance to hospital.	170,612 ambulance attendances to care homes over a 4 year period (2018 to 2021). A higher NEWS2 was associated with significantly increased conveyance to hospital (RRR 1.23, 95%CI 1.22–1.24, *p*<0,001)	Higher NEWS2 resulted in significantly increased emergency conveyance to hospital.
Voyer 2015 [37]	Peer-reviewed journal	Canada	Nursing home (*n*=1)	‘Validation study’	RADAR– an acute delirium screening tool.	Quantitative– sub-group analysis for nursing home residents re: sensitivity and specificity Survey– Feasibility questions but no sub-group analysis specifically for care home residents	Sensitivity decreased for residents of nursing home residents, compared to other groups. The prevalence of delirium for nursing home resident was low so caution required when interpreting sensitivity and specificity. Residents (nursing home), (*n*=40) Sensitivity % [95% CI] = 100.0 [2.5-100.0] Specificity % [95% CI] = 43.6 [27.8-60.4] Positive, predictive value, % [95% CI] = 4.3 [0.1-21.9] Negative, predictive value, % [95% CI] = 100.0 [80.5-100.0] Data were presented separately for the nursing home compared to patients receiving acute hospital care.	The RADAR tool is effective and accepted by nursing staff, and is an option for delirium screening in nursing homes.
Tingström 2015 [38]	Peer-reviewed journal	Sweden	Nursing homes (*n*=6)	(Validation study)	Early Detection Scale of Infection (EDIS)	Quantitative– relationship between the components of the EDIS for suspected infection, and the presence or absence of infection (determined by two physicians)	204 residents. Of 388 events of suspected infection, 49% were assessed as infection, 20% no infection and 31% as possible infection. EDIS instrument correctly predicted residents with ‘no infection’ and ‘infection’ in 67 and 84% of cases. However, it ‘did not have precision’ in predicting possible infection. Results also presented for the content validity of the 13 items of EDIS. Temperature, respiratory symptoms and ‘general signs of symptoms of illness’ were significantly related to infection.	In relation to the validation of EDIS, authors conclude that non-specific signs of deterioration (“general signs and symptoms of illness” and he/she is not as usual”) made by LTCF staff (nursing assistants) should be given high importance in the LTCF population.
Hockman-McDowell 2018 [33]	Conference abstract	USA	LTCFs (*n*=2)	‘Quality improvement, longitudinal study design’	Stop and Watch (from Interventions to Reduce Acute Care Transfers, INTERACT) Co-interventions– Nurse champions and staff education	Chart review 11 months pre-Stop and Watch and 3 months post (staff survey of skills acquired not included)	‘Reporting patient change pre and post Stop and Watch statistically significant (Z = -3.000, *p* = 0.003).’ Comparisons were made pre and post-Stop and Watch: - ‘reduction of falls 90.4%.’ - decreased falls with injury (Z = -2.840, *p* = 0.005) - ‘paired samples t-test decreased falls, pre mean 0.7753 (SD 1.42) to post mean 0.0851 (SD 0.28)’– ‘statistically and clinically significant (t(93) = 4.610, *p* = 0.000).’ - ‘positive correlation with statistical significance ER visits decreased (*n* = 94, correlation 0.308, *p*=.003).’’	Stop and Watch INTERACT improved staff ability to report resident changes. Decreases in falls leading to injury and unplanned hospital transfer were observed.
ElBestawi 2018 [43]	Peer-reviewed journal	Canada	Pilot site 1 - one long-term care facility (LTCF) Pilot site 2 - 4 LTCFs	‘Pilot study’ (mixed methods service evaluation)	Practical Routine Elder Variants Indicate Early Warning for Emergency Department (PREVIEW-ED)– focusing specifically on pneumonia, urinary tract infection, congestive heart failure and dehydration.	Quantitative– including rate of ’tool-sensitive transfers.’ Feedback from LTCF staff and healthcare professionals.	Pilot site 1 (P1), one facility, *n*=66 Pilot (P2), four facilities, *n*=176 >> Decrease in tool- sensitive transfers P1 = 57% P2 = 71% >> Tool completion rate P1 = 95.5% P2 = 94% >> Average time to complete tool per resident P1 = 8-15 seconds P2 = 10 seconds >> Average number of residents triggering tool/week P1 = 1 in 10 P2 = 1 in 20 >> Number of residents triggering the tool at least once P1 = 53% P3 = 37% Feedback (outside of a qualitative interview): >> LTCF staff feedback was positive: 1/ Staff feeling more valued, and having a ‘voice.’ 2/ Helps LTCF to judge when discussion with healthcare providers is necessary. >> Physicians perceived that PREVIEW-ED gave staff a better ‘grasp’ of the deterioration scenario, but did not increase the number of calls to healthcare providers. >> Management staff described enhancing communication with families. >> Unintended benefits, 1/ helps for deteriorations due to other causes e.g. in an Influenza outbreak, 2/ more time for staff to inform families of a dying trajectory >> The ’amount of paper generated,’ as the tool was completed daily, was highlighted as a drawback.	The deterioration tool has had a positive impact in reducing hospital transfers (for the four specific conditions). The authors suggest that this is likely to improve quality of life. A provisional economic evaluation suggests potential benefit.
Teale 2018 [35]	Peer-reviewed journal	UK	Residential and nursing care homes (*n*=9)	‘Prospective observational study’	Delirium Observational Screening Scale (DOSS)	Quantitative– diagnostic test accuracy and test–retest reliability of the DOSS to detect delirium (as part of routine care) in LTCFs.	216 residents participated. Half of the expected number of DOSS assessments occurred (30,201), out of which 11,659 (39%) were complete. ‘A cut point of 5 or more on the DOSS maximised sensitivity (0.61 95% CI: 0.39–0.80) and specificity (0.71 95% CI: 0.70–0.73); area under the ROC was 0.66 (95% confidence interval 0.58–0.80).’ Inter-rater reliability ‘good’ (ICC = 0.71, 95% CI: 0.61–0.78). Positive and negative predictive values 1.6 and 99.5% respectively.	Routine administration of the DOSS by care home staff was feasible. The 25-item DOSS has low sensitivity, limiting its clinical utility, but ‘acceptable’ specificity for delirium detection in care homes.
Little 2019 [28]	Peer-reviewed journal	UK	Care home, residential facility) (*n*=1)	‘Quality improvement project’ (before-after study)	Significant 7 early warning tool Co-intervention - training programme about deterioration recognition. Training sessions used the PDSA (plan, do, study, act) cycle.	Survey - Staff confidence Quantitative - Falls incidence Pressure ulcer incidence	Staff questionnaire at three time points: 12 reported using Significant 7 daily, 3 at least weekly. 22 residents participated. Falls frequency before and after intervention. The authors presented graphs showing the incidence of pressure sores and falls per week, at different points within the implementation / PDSA cycle.	Tool introduction resulted in positive benefits for staff and residents. Following Significant 7 introduction, there was a reduction in pressure ulcer and falls frequency. Staff reported increased confidence in responding to resident deterioration.
Tingstrom 2023 [42]	Peer-reviewed journal	Sweden Nursing home (*n*=1)– different cohort than in Tingström et al. 2015^22^		‘Longitudinal cohort exploratory design’	Early Detection Scale of Infection (EDIS), composed of 12 domains.	Quantitative– outcome = infection verified by GP +/- c-reactive protein. Factor analysis and logistic regression.	45 residents– 15 diagnosed with infection, 31 experienced 72 events of suspected infection. 189 observations were recorded from these events. Factor analysis 1: five components (‘change on cognitive/physical function,’ ‘general signs of symptoms of illness,’ ‘increased tenderness,’ ‘change in expression/food intake,’ ‘change in emotions’) explained 61% of the of the variance. Factor analysis 2, included dichotomous variable of temperature >1.0 °C from baseline temperature: five components (‘change in physical function/food intake,’ ‘confusion/signs and symptoms from respiratory/urinary tract,’ ‘general signs/symptoms of illness and fever,’ ‘increased tenderness,’ and ‘change in emotions’) explained 59% of the variance. From 72 episodes of suspected infection, 2 logistic regressions using the components from the 2 factor analyses conducted (infection the primary outcome): - Multivariate logistic regression 1: statistically significant as a model (*p*=0.032), with 2 components statistically significantly associated with infection– ‘increased tenderness’ (*p*=0.008) and ‘change in eye expression and food intake’ (*p*=0.008). - Multivariate logistic regression 1: overall *p*-value was not statistically significant (*p*=0.109) but 3 components were statistically significantly associated with infection– ‘change in physical function and food intake’ (*p*=0.022), ‘general signs and symptoms of illness and fever’ (*p*=0.018), and ‘increased tenderness (*p* > 0.012).	The EDIS tool has the potential to aid first-line caregivers to assess health deteriorations, helps to standardise communication and ensure that decisions are ‘not being taken at the wrong level.’ The purpose of EDIS is to act as a decision support tool, to ‘facilitate the step before diagnosis,’ not to determine medical treatment. Fever of >1.0°C (higher than baseline temperature for the individual) is a valuable parameter to add into the EDIS tool.

**Table 2:** Summary table (*unplanned hospital attendance incorporates ED attendance and hospital admission)

**Publication author, year**	**Peer-reviewed publication **	**Deterioration tool**				**Co-interventions**	**Quantitative outcomes** (unplanned emergency hospital attendance*)	**Data from qualitative interviews**		**Implementation challenges described**	**Authors conclude potential benefit**
		** National Early warning score**	**SBAR**	**STOP AND WATCH**	**Other**			**LTCF staff perspective**	**Healthcare staff perspective**		
O’Neill 2017 [41]	X		X		X	X		X		X	X
Russell 2020 [22]	X	X	X					X	X	X	X
Sampson 2020 [45]	X		X	X		X	X			X	
Stocker 2021 [17]	X	X	X			X		X	X	X	X
Porter 2021 [29]	X		X	X	X	X	X (X)				X
Montgomery 2023 [24]	X		X		X		X				X
Basinska 2022 [27]	X		X	X	X	X	X	X		X	X
Nwolise 2024 [44]	X	X	X		X		X	X		X	X
Renz 2013 [31]	X		X				X				X
Renz 2015 [32]	X		X				X (X)			X	X
Jarboe 2015 [23]			X				X (X)				
Ashcraft 2017 [30]	X		X				X (X)	X		X	X
Devereaux 2016 [34]			X				X (X)				X
Owen 2019 [36]	X		X					X	X		X
Luna 2023 [40]	X		X					X	X	X	X
Barker 2019 [25]	X	X					X				X
Stow 2021 [26]	X	X					X				X
Hodgson 2022 [21]	X	X				X	X (X)	X			X
Siriwardena 2024 [39]	X	X					X (X)				
Voyer 2015 [37]	X				X		X				X
Tingstrom 2015 [38]	X				X		X				X
Hockman-McDowell 2018 [33]				X			X (X)				X
ElBestawi 2018 [43]	X				X		X			X	X
Teale 2018 [35]	X				X		X				
Little 2019 [28]	X				X	X	X				X
Tingstrom 2023 [42]	X				X		X				X

Nine studies evaluated an intervention incorporating more than one deterioration tool [17, 22, 24, 25, 27, 29, 41, 44, 45], and/or deterioration tool use as part of a multi-component intervention (*n* = 8) [17, 21, 27–29, 33, 41, 45] such as additional personnel [27, 41], training beyond education specific to deterioration tool implementation [28, 29, 33] or enhanced clinical care pathways [45].

### Deterioration tool description and categorisation

Overall, deterioration tools aimed to facilitate, 1) the prompt recognition of acute deterioration by providing a system for care staff to, (a) observe changes in resident condition that may indicate deterioration [24, 27–29, 33, 35, 37, 38, 41–45] (including delirium screening tools [35, 37]) and/or, (b) by measuring vital signs as an objective measure of acute illness severity [17, 21, 22, 24–26, 29, 39, 42, 44], and/or 2) structured communication by LTCF staff with external healthcare professionals about acute deterioration [23, 24, 27, 30–32, 34, 36, 40, 41, 44, 45]. Deterioration tools are targeted towards the care staff response to acute deterioration, as opposed to supporting healthcare staff in managing acute illness.

SBAR is a communication tool, widely used in the hospital setting, providing a structure (situation-background-action-recommendation) to improve communication between care providers [46]. Two studies described tools based on SBAR [24, 34], including the Clinical Handover Assessment Tool (CHAT) which was used in conjunction with the Residential Aged Care Facility Emergency Decision Index (REDI) tool [24]. The REDI is a clinical decision guide for LTCF staff, which uses a combination of vital sign measurement and care staff observations (such as changes in resident activity level) to guide an escalation plan. This tool consists of 10 scenarios, including respiratory distress, urinary tract infection and delirium.

The National Early Warning Score (NEWS), modified to NEWS2 in 2017 [16], is advocated by UK policy makers for adoption in care homes [25]. NEWS is a standardised system initially designed for use in hospitals to recognise and communicate about acute illness. It is proposed that NEWS can act as a ‘common language’ for sharing information about resident deterioration between UK care homes and primary care/emergency services [16]. NEWS requires the measurement of temperature, pulse, systolic blood pressure, respiratory rate, oxygen saturation and level of consciousness. The overall NEWS triggers a response, ranging from continued monitoring to emergency service involvement [16].

Other tools captured changes in resident health and wellbeing, which would be observed by LTCF staff in their daily interactions with residents, as opposed to a reliance on the measurement of vital signs. STOP AND WATCH is used when a person is ‘not their usual self.’ There are 12 categories of observations such as ‘seeming different to usual’ (S), talking less (T), overall needing more help (O), and pain (P) [47]. The Significant Seven tool addresses seven similar signs of deterioration - confusion, mood, pain, hydration, skin, breathing changes and bowel habit [48]. The EDIS tool is the subject of two studies [38, 42]. This tool also uses changes in resident status observed by carers, such as confusion and changed appetite, which may indicate acute illness. The single vital sign of temperature is also included. The PREVIEW-ED [43] tool prompts Personal Support Workers to identify whether their resident is their ‘normal’ self, and the score indicates whether registered staff should be informed. The tool then prompts action by registered staff to complete an assessment and undertake further actions. RESTORE2 (Recognise Early Soft Signs, Take Observations, Respond, Escalate) [44] is a composite deterioration tool, incorporating the 1) ‘soft signs’ of deterioration (informal observation by care staff about a deterioration in resident health, 2) NEWS2, 3) SBARD (situation-background-assessment-recommendation-decision).

All but five [29, 34, 38, 42, 43] studies described a tool intended to assess all causes of acute deterioration– three studies focused on infection/sepsis identification [29, 38, 42] and two studies on specific ‘conditions’ [34, 43] resulting in deterioration. Two tools specifically aim to assist LTCF staff in identifying signs of acute delirium, which is an important sign of acute deterioration in older adults [49] - the Delirium Observational Screening Scale (DOSS) [35], and Recognizing Acute Delirium As Part Of Your Routine [RADAR] [37].

**Fig. 2:**
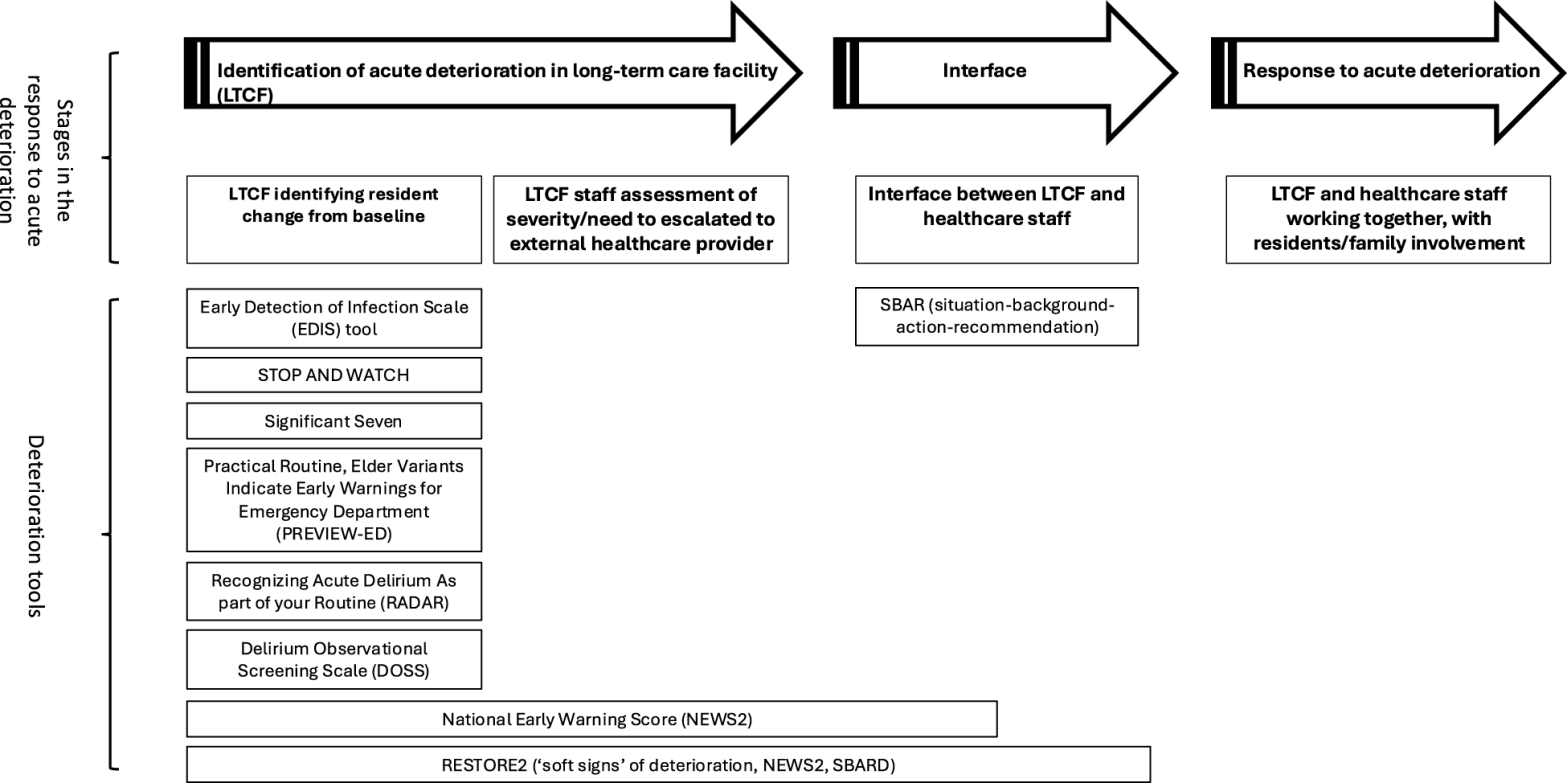
The process of responding to acute deterioration and at which stage deterioration tools are intended to act

The main point at which the deterioration tools are intended to act in the process of responding to acute resident deterioration tool is shown in Fig. 2. The majority of tools aim to assist LTCF staff to detect early signs of acute deterioration, which may be subtle. NEWS2 also aims to assist LTCF staff in assessing illness severity, and to act as a ‘common language’ [16] when communicating concerns with healthcare staff. SBAR, the most frequently used tool, is the only deterioration tool that is designed specifically to facilitate the exchange of information between LTCF and healthcare staff. RESTORE2 [44], as a composite deterioration tool, aims to act across multiple stages of the process. None of the deterioration tools identified were designed to be used jointly by healthcare and LTCF staff to respond to acute deterioration.

### How deterioration tools have been evaluated

The majority of studies (*n* = 21) used quantitative outcomes [21, 23–35, 37–39, 42–45] to evaluate deterioration tool use, with nine studies using qualitative outcomes [17, 21, 22, 27, 30, 36, 40, 41, 44] (excluding surveys with free-text responses [31] or feedback outside of a formal qualitative interview [43]. Only four studies [21, 27, 30, 44] used a combination of these outcomes. There was a high degree of heterogeneity in quantitative outcomes measured; unplanned hospital attendance was the commonest outcome measured [21, 23, 29, 30, 34, 35, 39, 43] (*n* = 8). Only three studies aimed to collect economic evaluation data [32, 43, 45]. The most frequent source of qualitative data was from LTCF staff [17, 21, 22, 30, 36, 40, 41, 44], with only four studies collecting data from healthcare staff in separate qualitative interviews [17, 22, 36, 40]. None of the studies assessed the impact of deterioration tools on residents’ (or relatives’) experience of being acutely unwell. Only one study attempted to collect quality of life data, but there was insufficient uptake of the intervention (incorporating STOP AND WATCH and SBAR tools) [45] to assess impact.

### Reported impact of deterioration tools

All but four [23, 27, 35, 45] of the included studies reported or concluded potential benefit from deterioration tool use. The most frequently reported benefit was increased staff confidence in managing acute deterioration [17, 21, 22, 24, 27, 28, 41, 44], including aiding communication about acute illness [17, 21, 22, 31, 36, 40, 44]. None of the papers reported significant negative impacts of tool use on LTCF care. However, implementation challenges of deterioration tools were highlighted in a significant proportion of studies in which qualitative data were collected (*n* = 10) [17, 22, 27, 30, 32, 40, 41, 43–45]. Important examples included deficiencies in the training on deterioration tools, staffing shortages in LTCFs, and the carer time required to use deterioration tools. Most notably, one paper described the abandonment of a cluster randomised study as no LTCFs (UK care homes in this case) adopted the complex intervention (incorporating two deterioration tools), which was aiming to decrease avoidable hospitalisation [45].

## Discussion

This review demonstrated that a variety of deterioration tools are being implemented in LTCFs, across a wide range of health and social care systems worldwide. Important gaps in the evidence base have been identified, limiting the assertions that can be made about deterioration tool use in this setting.

The most widely used deterioration tools are based on interventions transferred from the hospital setting, such as SBAR and NEWS, whilst others have been designed for use in LTCFs, such as STOP AND WATCH and the EDIS tool. It is commonplace for deterioration tools to be used in conjunction with one another, other co-interventions, or as part of wider programmes of care, as described in a previous review [15]. This limits the reliability of assertions that can be made about the specific impact of individual tools.

This review identifies SBAR as the most frequently used deterioration tool in LTCFs. SBAR, generally categorised as a deterioration tool, aims to structure communication between health and care providers. Hence, it is often used in conjunction with another tool. In the hospital setting, there is an established evidence base showing that SBAR improves communication between healthcare providers and improves patient safety [50]. The same assertions could not be made for the LTCF setting on the basis of evidence in this review.

The overriding majority of studies reported or concluded potential positive impact of deterioration tool use in LTCFs, offering evidence that deterioration tools are generally acceptable by LTCFs teams, and can increase confidence in responding to acute deterioration. There is some evidence to suggest that LTCF staff perceive that deterioration tools, especially SBAR, aid communication with external healthcare professionals. However, there was insufficient evidence to know if this matches the perspective of healthcare staff. Some studies suggested that deterioration tools may be associated with decreases in unplanned hospital transfer (but without assessment of appropriateness), but these studies did not have robust study designs, and were conducted across small numbers of LTCFs.

Although it was not within the specific aims of our scoping review (or the individual studies retrieved), the implementation challenges associated with deterioration tools were frequently described. The competing demands on care home staff are an important barrier to implementation. Failure to address implementation challenges in future work may result in inconsistent uptake of deterioration tools in LTCFs.

### Comparison with other work

Although there is a paucity of evidence about managing acute deterioration in care homes, our review builds on the findings of other studies [15, 18], and supports the assertion that this is a complex topic area. Our findings align with a previously published scoping reviewing showing that deterioration tools have frequently been evaluated as part of a multi-faceted model of care in Residential Aged Care settings [15]. This creates challenges in understanding the individual effects of the components of such interventions – the “black box effect” [51].

### Limitations

This review has identified important gaps in the evidence base, meaning that there is not sufficiently strong evidence to support the use deterioration tools in LTCFs. Most notably, the impact on the wellbeing and quality of life for residents has not been evaluated, meaning that there is insufficient evidence to know if tools improve resident care outcomes. The impact on community healthcare and emergency services is also unknown. Despite most authors concluding the positive impact of deterioration tools, especially LTCF staff confidence in managing acute illness, it is not known if deterioration tools facilitate staff and healthcare professionals to deliver proactive care, according to resident wishes. Within the body of evidence, there was insufficient information to understand the potential unintended consequences of deterioration tool use, such as the time taken for LTCF carers to use these tools [52].

The study designs that make up the body of evidence also limit the strength of the conclusions that can be drawn. Quality appraisal was not undertaken given the scoping nature of this work, but studies were low on the Hierarchy of Evidence. The majority of studies were conducted at a small number of LTCFs, with nearly half of included studies being single-site. This is a key deficit in the evidence base given the unique context of individual care facilities, and the potential implementation challenges that the studies in this review highlight. Studies in the current evidence synthesis did not have sufficiently long follow-up timeframes to know if deterioration tool uptake is sustainable in the longer term. Furthermore, the body of literature for deterioration tool use in LTCFs has a high degree of heterogeneity; studies have employed a wide range of methodologies, and there is little consistency in the outcomes used to evaluate the impact of tool use.

Grey literature searching did not identify any deterioration tools which had not already been identified during database searching. However, grey literature searching was confined to known relevant sources, as opposed to a wider searching strategy. In addition, four studies published by members of the authorship team were identified, relating to the use of NEWS in the same region of England.

### Implications

The gaps in the evidence base mean that no specific tool has been shown to improve resident care, but tools that support carer judgement about acute deterioration should be encouraged. There is evidence that LTCF staff find SBAR acceptable and its use intuitive, so this should be prioritised for future development and evaluation. The overriding majority of tools used in the retrieved studies aim to assist carers in identifying the subtle signs of deterioration and/or communicate these concerns to healthcare professionals (using the SBAR tool). Future tool development should focus on helping LTCF staff to assess illness severity and to judge how/when to escalate their concerns to external healthcare providers.

Further research is required to explore the experiences of acute deterioration for residents living in LTCFs, to measure the impact of tools on resident experience/health outcomes and to understand if deterioration tools align with their priorities of care. Further studies that explore the perspectives of healthcare professionals of interacting with LTCFs to manage acute deterioration are also required.

Implementation challenges and inconsistent deterioration tool uptake, both across LTCFs and within individual facilities, is a challenge to be considered in future work. There are important differences in staff skillset across different LTCFs in different settings. To ensure the adoption, implementation, and long-term effectiveness of tools, the unique context of individual LTCFs must be appreciated, and tools are required to be adaptable to the needs of individual LTCFs and the healthcare services they interact with. Tools transferred from the hospital setting should be adapted to the LTCF setting. Implementation theory and frameworks would help to ensure successful adoption of tools in LTCFs, and to ensure that the uptake is not inconsistent across the LTCF sector.

This review makes an important contribution to the evidence base about managing acute deterioration, and specifically about the use of deterioration tools and how they have been evaluated. This is an important step in helping to ensure that the potential spread of deterioration tools is grounded in evidence-based good practice, by informing a future systematic review or studies of effectiveness. The benchmark for investigating the effectiveness of deterioration tools would be randomised study designs that allow tools to be compared to standard care. However, given the challenges of this approach in LTCFs and the accelerated introduction of deterioration tools in this sector, ‘natural experiments’ [53] may be a suitable approach.

## Conclusion

This scoping review has identified emerging evidence that deterioration tools may have a role in assisting staff in LTCFs to identifying acute deterioration. Important evidence gaps, and limitations in the nature of the evidence base, mean that direct benefits for resident care have not been demonstrated. Despite strong policy drivers advocating the use of deterioration tools in LTCFs, there is not currently robust evidence to support the use of deterioration tools in LTCFs. This should be a focus for future research.

## Supplementary Information

Supplementary Material 1.

## Data Availability

Data sharing is not applicable to this article as no datasets were generated or analysed during the current study.
