# Supplementary material for: Which acute deterioration tools are used in long-term care facilities and how have they been evaluated? A scoping review
Source: BMC Health Serv Res. 2025 May 28;25:765. doi: 10.1186/s12913-025-12534-x (PMC12117722; doi:10.1186/s12913-025-12534-x)
Supplement: Supplementary file 1 — Supplementary Material 1. [file 12913_2025_12534_MOESM1_ESM.docx]

**Supplementary material – database searches**

Contents

[Summary 1](#_Toc131577400)

[Medline 2](#_Toc131577401)

[EMBASE 4](#_Toc131577402)

[PSYCINFO 6](#_Toc131577403)

[HMIC 7](#_Toc131577404)

[CINHAL 9](#_Toc131577405)

# Summary

Search date: 04 April 2023

| Database | No. of Results |
| --- | --- |
| Medline | 1102 |
| EMBASE | 1651 |
| PsycInfo | 274 |
| HMIC | 199 |
| CINHAL | 3179 |
|  |  |
| Total before deduplication | 6405 |
| Total after deduplication | 5270 |

# Medline

Database(s): **Ovid MEDLINE(R) and Epub Ahead of Print, In-Process, In-Data-Review & Other Non-Indexed Citations, Daily and Versions**1946 to April 03, 2023
Search Strategy:

| **#** | **Searches** | **Results** |
| --- | --- | --- |
| 1 | Long-Term Care/ | 28305 |
| 2 | Housing for the Elderly/ | 1652 |
| 3 | Homes for the Aged/ | 14730 |
| 4 | Assisted Living Facilities/ | 1586 |
| 5 | Nursing Homes/ | 39052 |
| 6 | Residential Facilities/ | 5737 |
| 7 | ((Longterm or long term) adj3 (care or facility or facilities)).ti,ab,kw. | 30358 |
| 8 | ((Nursing or resident* or group) adj3 home*).ti,ab,kw. | 46051 |
| 9 | (community adj1 care).ti,ab,kw. | 7302 |
| 10 | (residential care or residential facilit*).ti,ab,kw. | 5429 |
| 11 | (Healthcare facilit* or assisted living facilit* or assisted facilit*).ti,ab,kw. | 9284 |
| 12 | ("care home*" or care home setting* or care home service* or care home sector*).ti,ab,kw. | 5451 |
| 13 | ((geriatric or elder or aged) adj3 (facilit* or residen* or care home)).ti,ab,kw. | 8950 |
| 14 | out-of-hospital setting*.mp. | 497 |
| 15 | or/1-14 | 135130 |
| 16 | ("NEWS" or "NEWS2").ti,ab,kw. | 28374 |
| 17 | ("National Early Warning Score*" or "Individual Early Warning Score*").mp. | 675 |
| 18 | "Early Warning Score*".ti,ab,kw. | 1652 |
| 19 | "RESTORE2".ti,ab,kw. | 1 |
| 20 | "Stop and Watch".ti,ab,kw. | 106 |
| 21 | ("SBAR" or "SBARD").ti,ab,kw. | 266 |
| 22 | "Significant Seven".ti,ab,kw. | 103 |
| 23 | ("track and trigger" or "track and trigger system*" or "early warning score*" or "severity of illness index").mp. | 271753 |
| 24 | "clinical assessment*".mp. | 36309 |
| 25 | clinical tool.mp. | 6226 |
| 26 | exp "Severity of Illness Index"/ | 280379 |
| 27 | exp Patient Handoff/ | 1569 |
| 28 | ((deteriorat* or escalat*) adj3 tool*).mp. | 97 |
| 29 | (tool* adj25 "bedside evaluation").mp. | 24 |
| 30 | or/16-29 | 351594 |
| 31 | 15 and 30 | 2774 |
| 32 | limit 31 to yr="2013 -Current" | 1102 |

# EMBASE

Database(s): **Embase**1974 to 2023 April 03
Search Strategy:

| **#** | **Searches** | **Results** |
| --- | --- | --- |
| 1 | long term care/ | 145064 |
| 2 | home for the aged/ | 11438 |
| 3 | assisted living facility/ | 3191 |
| 4 | nursing home/ | 61091 |
| 5 | residential home/ | 7824 |
| 6 | ((Longterm or long term) adj3 (care or facility or facilities)).ti,ab,kw. | 39937 |
| 7 | ((Nursing or resident* or group) adj3 home*).ti,ab,kw. | 59785 |
| 8 | (community adj1 care).ti,ab,kw. | 9259 |
| 9 | (residential care or residential facilit*).ti,ab,kw. | 6837 |
| 10 | (Healthcare facilit* or assisted living facilit* or assisted facilit*).ti,ab,kw. | 12758 |
| 11 | ("care home*" or care home setting* or care home service* or care home sector*).ti,ab,kw. | 7385 |
| 12 | ((geriatric or elder or aged) adj3 (facilit* or residen* or care home)).ti,ab,kw. | 11165 |
| 13 | out-of-hospital setting*.mp. | 764 |
| 14 | or/1-13 | 282725 |
| 15 | national early warning score/ | 432 |
| 16 | ("NEWS" or "NEWS2").ti,ab,kw. | 30138 |
| 17 | ("National Early Warning Score*" or "Individual Early Warning Score*").mp. | 1199 |
| 18 | "Early Warning Score*".ti,ab,kw. | 2688 |
| 19 | "RESTORE2".ti,ab,kw. | 4 |
| 20 | "Stop and Watch".ti,ab,kw. | 194 |
| 21 | ("SBAR" or "SBARD").ti,ab,kw. | 514 |
| 22 | "Significant Seven".ti,ab,kw. | 128 |
| 23 | ("track and trigger" or "track and trigger system*" or "early warning score*" or "severity of illness index").mp. | 24558 |
| 24 | "clinical assessment*".ti,ab,kw. | 55733 |
| 25 | clinical tool.ti,ab,kw. | 9364 |
| 26 | exp "severity of illness index"/ | 20388 |
| 27 | exp clinical handover/ | 10113 |
| 28 | ((deteriorat* or escalat*) adj3 tool*).mp. | 174 |
| 29 | (tool* adj25 "bedside evaluation").mp. | 48 |
| 30 | or/15-29 | 129434 |
| 31 | 14 and 30 | 2164 |
| 32 | limit 31 to yr="2013 -Current" | 1651 |

# PSYCINFO

Database(s): **APA PsycInfo**1806 to March Week 4 2023
Search Strategy:

| **#** | **Searches** | **Results** |
| --- | --- | --- |
| 1 | Long Term Care/ | 6381 |
| 2 | Assisted Living/ | 850 |
| 3 | Nursing Homes/ | 9724 |
| 4 | Residential Care Institutions/ | 11401 |
| 5 | ((Longterm or long term) adj3 (care or facility or facilities)).ti,ab. | 9601 |
| 6 | ((Nursing or resident* or group) adj3 home*).ti,ab. | 18446 |
| 7 | (community adj1 care).ti,ab. | 3372 |
| 8 | (residential care or residential facilit* or residential institut*).ti,ab. | 5620 |
| 9 | (Healthcare facilit* or assisted living facilit* or assisted facilit*).ti,ab. | 1427 |
| 10 | ("care home*" or care home setting* or care home service* or care home sector*).ti,ab. | 2215 |
| 11 | ((geriatric or elder or aged) adj3 (facilit* or residen* or care home)).ti,ab. | 3648 |
| 12 | out-of-hospital setting*.mp. | 15 |
| 13 | or/1-12 | 47517 |
| 14 | ("NEWS" or "NEWS2").ti,ab. | 16557 |
| 15 | ("National Early Warning Score*" or "Individual Early Warning Score*").mp. | 16 |
| 16 | "Early Warning Score*".ti,ab. | 56 |
| 17 | "Stop and Watch".ti,ab. | 86 |
| 18 | ("SBAR" or "SBARD").ti,ab. | 39 |
| 19 | "Significant Seven".ti,ab. | 18 |
| 20 | ("track and trigger" or "track and trigger system*" or "early warning score*" or "severity of illness index").mp. | 38167 |
| 21 | "clinical assessment*".mp. | 11597 |
| 22 | clinical tool.mp. | 1260 |
| 23 | ((deteriorat* or escalat*) adj3 tool*).mp. | 24 |
| 24 | (tool* adj25 "bedside evaluation").mp. | 4 |
| 25 | or/14-24 | 67211 |
| 26 | 13 and 25 | 924 |
| 27 | limit 26 to yr="2013 -Current" | 274 |

# HMIC

Database(s): **HMIC Health Management Information Consortium**1979 to January 2023
Search Strategy:

| **#** | **Searches** | **Results** |
| --- | --- | --- |
| 1 | Long term care/ | 1908 |
| 2 | old peoples homes/ | 494 |
| 3 | assisted community homes/ | 13 |
| 4 | nursing homes/ | 1639 |
| 5 | ((Longterm or long term) adj3 (care or facility or facilities)).ti,ab. | 2392 |
| 6 | ((Longterm or long term) and (care or facility or facilities)).hw. | 2040 |
| 7 | ((Nursing or resident* or group) adj3 home*).ti,ab. | 4116 |
| 8 | ((Nursing or resident* or group) and home*).hw. | 3788 |
| 9 | (community adj1 care).ti,ab. | 6382 |
| 10 | (community and care).hw. | 12670 |
| 11 | (residential care or residential facilit*).ti,ab. | 2431 |
| 12 | (residential care or residential facilit*).hw. | 4905 |
| 13 | (Healthcare facilit* or assisted living facilit* or assisted facilit*).ti,ab. | 242 |
| 14 | ("care home*" or care home setting* or care home service* or care home sector*).ti,ab. | 1900 |
| 15 | ("care home*" or care home setting* or care home service* or care home sector*).hw. | 1376 |
| 16 | ((geriatric or elder or aged) adj3 (facilit* or residen* or care home)).ti,ab. | 275 |
| 17 | ((geriatric or elder or aged) and (facilit* or residen* or care home)).hw. | 79 |
| 18 | out-of-hospital setting*.mp. | 3 |
| 19 | or/1-18 | 26623 |
| 20 | ("NEWS" or "NEWS2").ti,ab. | 862 |
| 21 | ("NEWS" or "NEWS2").hw. | 10 |
| 22 | ("National Early Warning Score*" or "Individual Early Warning Score*").mp. | 12 |
| 23 | "Early Warning Score*".ti,ab. | 29 |
| 24 | "Stop and Watch".ti,ab. | 2 |
| 25 | ("SBAR" or "SBARD").ti,ab. | 8 |
| 26 | "Significant Seven".ti,ab. | 1 |
| 27 | ("track and trigger" or "track and trigger system*" or "early warning score*" or "severity of illness index").mp. | 49 |
| 28 | ("track and trigger" or "track and trigger system*" or "early warning score*" or "severity of illness index").hw. | 12 |
| 29 | "clinical assessment*".mp. | 344 |
| 30 | "clinical assessment*".hw. | 34 |
| 31 | clinical tool.mp. | 35 |
| 32 | Residential care/ | 4393 |
| 33 | ((deteriorat* or escalat*) adj3 tool*).mp. | 3 |
| 34 | ((deteriorat* or escalat*) and tool*).hw. | 1 |
| 35 | or/20-34 | 5669 |
| 36 | 19 and 35 | 4446 |
| 37 | limit 36 to yr="2013 -Current" | 199 |

# CINHAL

Tuesday, April 04, 2023 10:46:40 AM

| **#** | **Query** | **Results** |
| --- | --- | --- |
| S24 | S10 AND S23 Limiters - Published Date: 20130101-20231231 | 3,179 |
| S23 | S11 OR S12 OR S13 OR S14 OR S15 OR S16 OR S17 OR S18 OR S19 OR S20 OR S21 OR S22 | 325,617 |
| S22 | (MH "Hand Off (Patient Safety)") | 2,757 |
| S21 | TX (tool* N25 "bedside evaluation" | 5 |
| S20 | TX ((deteriorat* OR escalat*) N3 tool*) | 89 |
| S19 | MH Severity of Illness Indices | 63,711 |
| S18 | MH clinical assessment tools | 195,724 |
| S17 | TX ("track and trigger" OR "track and trigger system*" OR "early warning score*" OR "severity of illness index") | 1,177 |
| S16 | (TI "Significant Seven" OR AB "Significant Seven") | 26 |
| S15 | ((TI "SBAR" OR AB "SBAR") OR (TI "SBARD" OR AB "SBARD")) | 318 |
| S14 | (TI "Stop and Watch" OR AB "Stop and Watch") | 8 |
| S13 | (TI "RESTORE2" or AB "RESTORE2") | 48 |
| S12 | ((TI "NEWS" OR AB "NEWS") OR (TI "NEWS2" OR AB "NEWS2")) | 68,222 |
| S11 | (MH "Early Warning Score") | 163 |
| S10 | S1 OR S2 OR S3 OR S4 OR S5 OR S6 OR S7 OR S8 OR S9 | 122,571 |
| S9 | TX "out-of-hospital setting*" | 328 |
| S8 | (((TI geriatric OR AB geriatric) OR (TI elder OR AB elder) OR (TI aged OR AB aged)) N3 ((TI facilit* OR AB facilit*) OR (TI residen* OR AB residen*) OR (TI "care home" OR AB "care home"))) | 6,075 |
| S7 | ((TI "care home*" OR AB "care home*") OR (TI "care home setting*" OR AB "care home setting*") OR (TI "care home service*" OR AB "care home service*") OR (TI "care home sector*" OR AB "care home sector*")) | 6,555 |
| S6 | ((TI healthcare facilit* OR AB healthcare facilit*) OR (TI assisted living facilit* OR AB assisted living facilit*) OR (TI assisted facilit* OR AB assisted facilit*)) | 15,040 |
| S5 | ((TI "residential care" OR AB "residential care") OR (TI "residential facilit*" OR AB "residential facilit*")) | 4,474 |
| S4 | ((TI community OR AB community) N1 (TI care OR AB care)) | 11,636 |
| S3 | (((TI Nursing OR AB Nursing) OR (TI resident* OR AB resident*) OR (TI group OR AB group)) N3 (TI home* OR AB home*)) | 35,635 |
| S2 | (((TI Longterm OR AB Longterm) OR (TI "long term" OR AB "long term")) N3 ((TI care OR AB care) OR (TI facility OR AB facility) OR (TI facilities OR AB facilities))) | 23,723 |
| S1 | MH long-term care or nursing home or residential care or assisted living | 74,289 |
